# Sitagliptin does not reduce the risk of cardiovascular death or hospitalization for heart failure following myocardial infarction in patients with diabetes: observations from TECOS

**DOI:** 10.1186/s12933-019-0921-2

**Published:** 2019-09-03

**Authors:** Michael A. Nauck, Darren K. McGuire, Karen S. Pieper, Yuliya Lokhnygina, Timo E. Strandberg, Axel Riefflin, Tuncay Delibasi, Eric D. Peterson, Harvey D. White, Russell Scott, Rury R. Holman

**Affiliations:** 1 Division of Diabetology, Medical Department I, St. Josef-Hospital (Ruhr-University), Bochum, Germany; 2 Division of Cardiology, University of Texas Southwestern Medical Center, Dallas, TX USA; 3 Duke Clinical Research Institute, Duke University School of Medicine, Durham, NC USA; 4 Helsinki University Hospital, University of Helsinki, Helsinki, Finland; 5 Center for Life Course Health Research, University of Oulu, Oulu, Finland; 6 Practise Internal Medicine/Diabetology, Husby, Germany; 7 Department of Internal Medicine, Hacettepe University, Ankara, Turkey; 8 Coronary Care and Cardiovascular Research at the Green Lane Cardiovascular Service, Auckland City Hospital, Auckland, New Zealand; 9 Don Beaven Medical Research Center, Christchurch Hospital, Christchurch, New Zealand; 10 Diabetes Trials Unit, Oxford Centre for Diabetes, Endocrinology and Metabolism, University of Oxford, Churchill Hospital, Oxford, UK

**Keywords:** Acute myocardial infarction, Cardiovascular outcomes, Sitagliptin, Type 2 diabetes

## Abstract

**Background:**

To examine the effects of the DPP-4i sitagliptin on CV outcomes during and after incident MI in the Trial Evaluating Cardiovascular Outcomes with Sitagliptin (TECOS).

**Methods:**

TECOS randomized 14,671 participants with type 2 diabetes and atherosclerotic cardiovascular disease (ASCVD) to sitagliptin or placebo, in addition to usual care. For those who had a within-trial MI, we analyzed case fatality, and for those with a nonfatal MI, we examined a composite cardiovascular (CV) outcome (CV death or hospitalization for heart failure [hHF]) by treatment group, using Cox proportional hazards models left-censored at the time of the first within-trial MI, without and with adjustment for potential confounders, in intention-to-treat analyses.

**Results:**

During TECOS, 616 participants had ≥ 1 MI (sitagliptin group 300, placebo group 316, HR 0.95, 95% CI 0.81–1.11, P = 0.49), of which 25 were fatal [11 and 14, respectively]). Of the 591 patients with a nonfatal MI, 87 (15%) died subsequently, with 66 (11%) being CV deaths, and 57 (10%) experiencing hHF. The composite outcome occurred in 58 (20.1%; 13.9 per 100 person-years) sitagliptin group participants and 50 (16.6%; 11.7 per 100 person-years) placebo group participants (HR 1.21, 95% CI 0.83–1.77, P = 0.32, adjusted HR 1.23, 95% CI 0.83–1.82, P = 0.31). On-treatment sensitivity analyses also showed no significant between-group differences in post-MI outcomes.

**Conclusions:**

In patients with type 2 diabetes and ASCVD experiencing an MI, sitagliptin did not reduce subsequent risk of CV death or hHF, contrary to expectations derived from preclinical animal models.

*Trial registration* clinicaltrials.gov no. NCT00790205

## Background

Dipeptidyl peptidase-4 inhibitors (DPP-4is) lower plasma glucose and glycated hemoglobin in people with type 2 diabetes by inhibiting degradation of endogenous glucagon-like peptide-1 (GLP-1) [1]. They have a low risk for hypoglycemia and are weight neutral [2]. Although two GLP-1 receptor agonists, once-daily liraglutide [3] and once-weekly semaglutide [4], have been shown to reduce cardiovascular (CV) events in patients with type 2 diabetes at high CV risk, four CV outcome trials that evaluated the once-daily DPP-4i agents saxagliptin [5], alogliptin [6], sitagliptin [7, 8], and linagliptin [9, 10] versus placebo showed no impact on CV death, myocardial infarction (MI), or stroke outcomes.

GLP-1 receptors are expressed on cells in CV tissues [11], and multiple CV effects of GLP-1 receptor agonism have been demonstrated with administration of native GLP-1, with administration of GLP-1 receptor agonists, and with DPP-4i treatment in preclinical studies [11–14]. Among these well-documented effects is a substantial (30–50%) reduction in the extent of myocardial necrosis after experimentally induced MI in rodents pretreated with native GLP-1 [15, 16] or with a GLP-1 receptor agonist [17, 18]. Similar experimental approaches with a DPP-4i in mice [19], rats [20], pigs [21], and dogs [22] produced largely similar results. Regarding potential mechanisms, sitagliptin seems to improve tolerance to ischemia as demonstrated by an improved regional contractility in ischemic segments of the left ventricle [23, 24]. These effects of DPP-4 inhibition may be mediated by protection of mitochondrial function and preventing cardiomyocyte apoptosis, and by interfering with oxidative stress during reperfusion [20, 21]. Theoretically, a smaller infarct size in humans could result in lower incident case-fatality, less post-MI arrhythmogenic risk, and higher residual left-ventricular function with a lower future risk of heart failure or CV death [25, 26].

The Trial Evaluating Cardiovascular Outcomes with Sitagliptin (TECOS) randomized patients with type 2 diabetes and atherosclerotic cardiovascular disease (ASCVD) to double-blind therapy with sitagliptin or placebo, in addition to usual care, aiming for glycemic equipoise [7, 8]. In a post hoc analysis, we evaluated the effects of sitagliptin on a composite outcome defined as CV death or hospitalization for heart failure (hHF) in TECOS participants who experienced a within-trial MI.

## Methods

### Study design

The TECOS design [8] and primary results [7] and heart failure outcomes [27] have been published previously. Briefly, 14,671 participants from 38 countries were enrolled between December 2008 and July 2012. Eligible participants were ≥ 50 years old (no upper age limit) with type 2 diabetes, ASCVD, and glycated hemoglobin (HbA_1c_) values of 6.5–8.0% (48–64 mmol/mol) on stable dose mono- or dual-combination therapy with metformin, pioglitazone, sulfonylurea or insulin (with or without metformin). Participants were randomized double-blind to sitagliptin or placebo at doses appropriate for their eGFR [7, 8]. During follow-up, treatment for hyperglycemia and for type 2 diabetes comorbidities was provided by usual care providers according to their local guidelines with addition of any open-label glucose-lowering agent permitted, apart from a GLP-1 receptor agonist or DPP-4i. All reported events of death, MI, stroke, and hospitalization for unstable angina or heart failure were adjudicated by an independent committee masked to randomized treatment assignment. Adjudicated event definitions have been published previously [7, 8].

### Objectives

The analyses presented here examine only those participants who experienced an MI during the trial. We evaluated potential differences between the randomized groups in case-fatality and for those with a non-fatal MI the time to a composite outcome defined as CV death or hHF. Secondary outcomes were post-MI time to CV death, hHF, and all-cause death. We also examined hHF in patients not known to have heart failure at baseline, and an extended composite outcome defined as CV death, hHF, a further MI, stroke, or new-onset atrial fibrillation.

### Statistical analysis

Baseline characteristics for continuous variables were summarized as median and interquartile range (IQR), and categorical variables as count (percentage).

Primary analyses were performed on the intention-to-treat population in the subset who experienced an MI during the trial. Secondary on-treatment sensitivity analyses were performed with participants classified as “DPP-4i treated” if they were taking double-blind sitagliptin study medication or if they were taking an open-label DPP-4i. Similarly, they were classified as “not DPP-4i treated” if they were taking double-blind placebo study medication or had discontinued double-blind sitagliptin study medication and were not taking an open-label DPP-4i.

The two treatment groups were compared using Cox proportional hazards models, without and with adjustment for potential confounders. Adjustment factors applied were those previously identified in the large Nateglinide and Valsartan in Impaired Glucose Tolerance Outcomes Research (NAVIGATOR) clinical trial [28, 29]. The assumptions of linearity and proportional hazards had been previously evaluated for the set of confounders considered and appropriate adjustments applied when violations were noted. The list of covariates is provided in Additional file 1: Table S1. The proportional hazards assumption was tested for the treatment factor in these new models, and time-varying models would have been applied had violations been noted. Follow-up began (day 0) at the date of the first within-trial MI and continued until the date of the first occurrence of each type of endpoint considered here or the date of last contact when no event occurred. The analyses were performed twice in consideration of fatal MIs. In one case (primary analyses), only patients with nonfatal MIs were considered; in the second, the fatal MIs were in the cohort and included as endpoints.

All analyses were performed using SAS version 9.4 (SAS Institute, Cary, NC).

## Results

### Participant characteristics

Baseline characteristics of all participants at entry to TECOS are listed in Table 1 according to whether or not they had experienced an MI. Those with, compared without, an MI were more likely to be male (77.9% vs. 70.4%, P < 0.0001), to have prior coronary artery disease (89.4% vs. 73.4%, P < 0.0001), prior MI (57.8% vs. 42.0%, P < 0.0001) or prior hHF (21.4% vs. 17.9%, P = 0.024); and to be treated less commonly with metformin (75.5% *vs.* 81.8%, P < 0.0001) and more commonly with insulin (33.5% vs. 22.8%, P < 0.0001).

**Table 1:** Baseline characteristics of TECOS participants who did not have a within-trial nonfatal myocardial infarction (MI), and for those participants with a nonfatal MI, split by sitagliptin or placebo treatment

Characteristic	Patients without a nonfatal MI during the trial randomized to sitagliptin or placebo N = 14,055	Patients with a nonfatal MI during the trial		*P*-value*
		Sitagliptin N = 289	Placebo N = 302	
Age at randomization (years)^a^	65.0 (60.0, 71.0)	67.0 (62.0, 74.0)	66.0 (60.0, 72.0)	0.1414
Female	4161 (29.6%)	56 (19.4%)	75 (24.8%)	0.1103
Hispanic or Latino	1754 (12.5%)	17 (5.9%)	22 (7.3%)	0.4924
Race				0.4603
White	9472 (67.4%)	235 (81.3%)	234 (77.5%)	
Black	422 (3.0%)	10 (3.5%)	14 (4.6%)	
Asian	3184 (22.7%)	38 (13.1%)	42 (13.9%)	
Other	977 (7.0%)	6 (2.1%)	12 (4.0%)	
Region				0.5023
Latin America	1445 (10.3%)	10 (3.5%)	11 (3.6%)	
Asia Pacific and other	4377 (31.1%)	98 (33.9%)	84 (27.8%)	
Western Europe	1977 (14.1%)	48 (16.6%)	50 (16.6%)	
Eastern Europe	3822 (27.2%)	63 (21.8%)	68 (22.5%)	
North America	2434 (17.3%)	70 (24.2%)	89 (29.5%)	
Duration^b^ of type 2 diabetes (years)	10.0 (5.0, 16.0)	11.0 (6.0, 18.0)	11.0 (6.0, 16.0)	0.3233
Diabetes therapy at baseline (alone or in combination)				
Sulfonylurea	6394 (45.5%)	110 (38.1%)	125 (41.4%)	0.4085
Metformin	11,501 (81.8%)	212 (73.4%)	235 (77.8%)	0.2069
Thiazolidinedione	376 (2.7%)	9 (3.1%)	9 (3.0%)	0.9245
Insulin	3208 (22.8%)	98 (33.9%)	98 (32.5%)	0.7063
Preexisting vascular disease	13,975 (99.4%)	288 (99.7%)	302 (100.0%)	0.4890
Coronary artery disease	10,312 (73.4%)	261 (90.3%)	269 (89.1%)	0.6208
Cerebrovascular disease	3445 (24.5%)	72 (24.9%)	65 (21.5%)	0.3289
Peripheral arterial disease	2348 (16.7%)	40 (13.8%)	42 (13.9%)	0.9814
Prior MI	5899 (42.0%)	171 (59.2%)	170 (56.3%)	0.4790
Prior congestive heart failure	2511 (17.9%)	62 (21.5%)	61 (20.2%)	0.7072
Previous atrial fibrillation/flutter	1086 (7.7%)	32 (11.1%)	45 (14.9%)	0.1670
NYHA classification				0.3241
1	510 (20.3%)	16 (25.8%)	8 (13.1%)	
2	1256 (50.0%)	24 (38.7%)	30 (49.2%)	
3	339 (13.5%)	6 (9.7%)	10 (16.4%)	
4	11 (0.4%)	1 (1.6%)	1 (1.6%)	
Not available	395 (15.7%)	15 (24.2%)	12 (19.7%)	
Qualifying HbA1c (mmol/mol)	55.2 (50.8, 60.7)	56.1 (51.0, 61.7)	55.2 (51.9, 59.6)	0.3239
Qualifying HbA1c (%)	7.2 (6.8, 7.7)	7.3 (6.8, 7.8)	7.2 (6.9, 7.6)	0.3239
eGFR (mL/min/1.73 m^2^)	73.0 (60.0, 88.0)	68.5 (55.0, 84.0)	69.0 (56.0, 88.0)	0.2918
Urine albumin creatinine ratio (g/mol creatinine)	10.6 (3.5, 35.0)	12.2 (5.3, 52.7)	13.8 (5.3, 43.9)	0.9026
Heart rate (bpm)	72.0 (65.0, 79.0)	70.0 (62.0, 78.0)	71.0 (62.0, 80.0)	0.0595
Body mass index (kg/m^2^)	29.5 (26.3, 33.2)	29.8 (26.6, 33.5)	30.4 (27.2, 34.3)	0.1480
Weight (kg)	83.0 (71.0, 96.0)	85.0 (75.0, 98.0)	88.5 (75.0, 100.0)	0.1517
Height (cm)	168.0 (160.0, 174.2)	169.4 (163.2, 175.3)	170.0 (162.6, 176.0)	0.5036
Cigarette smoking status				0.9049
Current	1589 (11.3%)	44 (15.2%)	43 (14.2%)	
Former	5575 (39.7%)	129 (44.6%)	133 (44.0%)	
Never	6891 (49.0%)	116 (40.1%)	126 (41.7%)	
Systolic blood pressure (mmHg)	133.0 (124.0, 145.0)	136.0 (124.0, 146.0)	135.0 (124.0, 148.0)	0.9803
Diastolic blood pressure (mmHg)	79.0 (70.0, 84.0)	77.0 (68.0, 82.0)	76.0 (68.0, 85.0)	0.3050
LDL-C	84.0 (65.0, 109.0)	81.0 (63.0, 99.6)	82.1 (65.6, 108.1)	0.4128
Medications taken at time of randomization				
Statins	11,213 (79.8%)	238 (82.4%)	248 (82.1%)	0.9408
ACE inhibitors or angiotensin receptor blockers	11,040 (78.5%)	238 (82.4%)	255 (84.4%)	0.4959
Diuretics	5727 (40.7%)	127 (43.9%)	150 (49.7%)	0.1633
Calcium channel blockers	4730 (33.7%)	104 (36.0%)	118 (39.1%)	0.4386
Beta blockers	8876 (63.2%)	210 (72.7%)	221 (73.2%)	0.8881
Aspirin	11,027 (78.5%)	244 (84.4%)	235 (77.8%)	0.0403

Data shown are median (interquartile range) or N (%)

*ACE* angiotensin-converting enzyme, *eGFR* estimated glomerular filtration rate, *HbA1c* glycated hemoglobin, *LDL-C* low-density lipoprotein cholesterol, *NYHA* New York Heart Association, *TECOS* Trial Evaluating Cardiovascular Outcomes with Sitagliptin

*P-value is for placebo vs sitagliptin in patients with a nonfatal MI

^a^Age is missing among patients enrolled in Lithuania because the entire birth date including year was not available

^b^Duration = (year of randomization − year of diagnosis) + 1

### Fatal and nonfatal MI

A total of 616 (4.2%) of the 14,671 TECOS participants had a within-trial fatal or nonfatal MI (300 [49%] randomized to sitagliptin and 316 [51%] to placebo), with no significant difference in the time to first event by randomized therapy (HR 0.95, 95% CI 0.81–1.11, P = 0.49) as reported previously [7]. Outcome information was missing for one participant for hHF and for two other participants for atrial fibrillation and stroke, limiting the number of participants who could be analyzed for these outcomes to 615 and 614, respectively. Twenty-five of these first MI events were fatal, 11 in the sitagliptin group and 14 in the placebo group, leaving 289 and 302 participants respectively with nonfatal MIs. Of the 591 participants who had a within-trial nonfatal MI, 87 (15%) died subsequently (66 [11%] classified as CV death), 57 (10%) experienced hHF, 109 (18%) had a second MI, 20 (3%) had a stroke, and 37 (6%) had incident atrial fibrillation.

### CV events after nonfatal MI

**Fig. 1:**
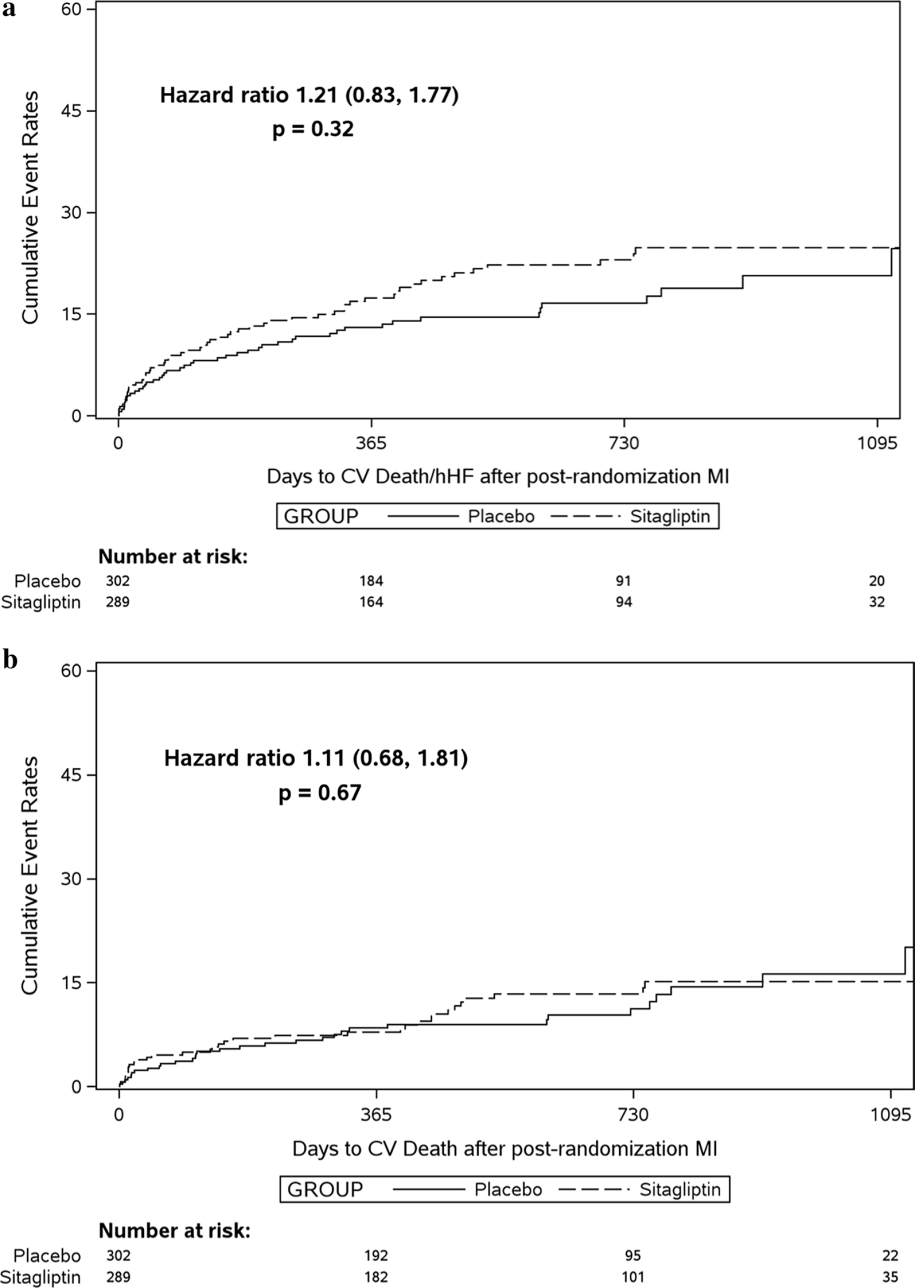
Unadjusted event curves by randomized assignment to sitagliptin or placebo (Kaplan–Meier plots) for the composite outcome of cardiovascular (CV) death or heart failure hospitalization (hHF) (**a**) and for CV death (**b**), both occurring after the first within-trial nonfatal myocardial infarction (MI) (defining day 0 on the x-axis). Intention-to-treat analysis

The composite outcome of CV death or hHF following a nonfatal MI occurred in 58 of 289 sitagliptin group participants (20.1%; 13.9 events per 100 person-years) and in 50 of 302 placebo group participants (16.6%; 11.7 per 100 person-years), with no significant difference between groups (HR 1.21, 95% CI 0.83–1.77, P = 0.32; adjusted HR 1.23, 95% CI 0.83–1.82, P = 0.31) (Fig. 1a and Table 2). Similar results were seen for the individual outcomes of CV death, hHF, incident heart failure, recurrent MI, and all-cause death, and for the extended composite (CV death, hHF, incident heart failure, recurrent MI, stroke, or incident atrial fibrillation), with no significant differences also seen after adjustment for potential confounders (Table 2). Results were also similar when fatal MI was included in the cohort of interest (Additional file 1: Table S2, Figure S1).

**Table 2:** Cardiovascular outcomes occurring after a first within-trial non-fatal myocardial infarction in those randomized previously to sitagliptin or placebo treatment (intention-to-treat analysis)

	Sitagliptin n = 289		Placebo n = 302		Unadjusted hazard ratio (95% CI)	P-value	Adjusted hazard ratio (95% CI)	P-value
	No. (%)	Events per 100 patient-years	No. (%)	Events per 100 patient-years				
Cardiovascular death or hospitalization for heart failure	58 (20.1)	13.9	50 (16.6)	11.7	1.21 (0.83–1.77)	0.32	1.23 (0.83–1.82)	0.31
Cardiovascular death	34 (11.8)	7.6	32 (10.6)	7.1	1.11 (0.68–1.81)	0.67	1.12 (0.67–1.86)	0.67
Hospitalization for heart failure	31 (10.7)	7.5	26 (8.6)	6.1	1.26 (0.75–2.12)	0.39	1.40 (0.80–2.42)	0.23
New onset heart failure	19 (6.6)	4.3	17 (5.6)	3.8	1.25 (0.64–2.44)	0.51	1.49 (0.72–3.09)	0.28
Cardiovascular death, hospital admission for heart failure, new heart failure, acute myocardial infarction, stroke or new-onset atrial fibrillation	108 (37.4)	33.0	100 (33.1)	28.4	1.16 (0.89–1.53)	0.27	1.21 (0.91–1.60)	0.20
Further acute myocardial infarction	54 (18.7)	7.4	55 (18.2)	7.1	1.01 (0.69–1.48)	0.95	0.99 (0.67–1.46)	0.95
All-cause death	50 (17.3)	11.0	37 (12.3)	8.1	1.40 (0.92–2.15)	0.12	1.41 (0.90–2.21)	0.13

### On-treatment sensitivity analyses

**Fig. 2:**
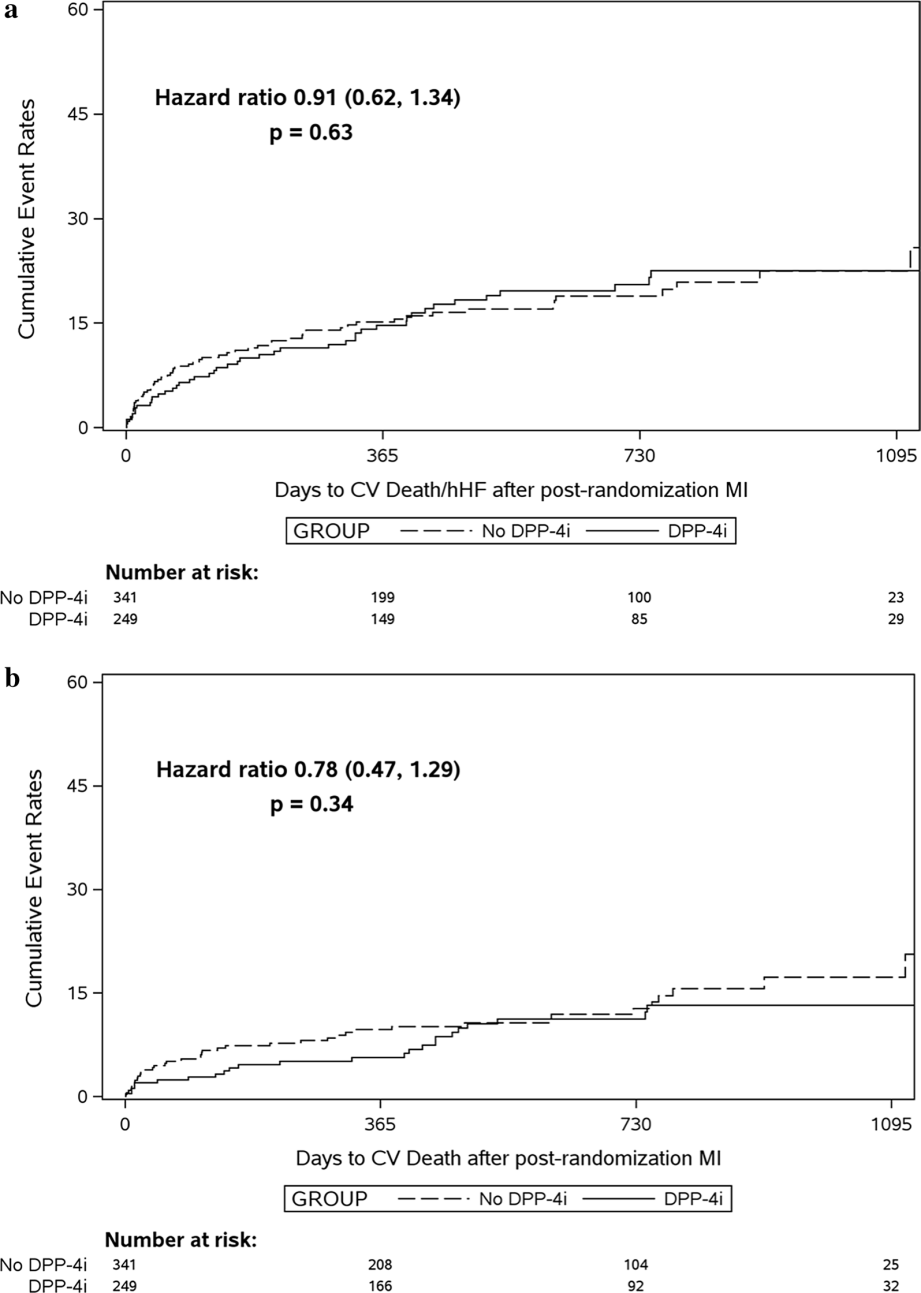
Unadjusted event curves by dipeptidyl peptidase-4 inhibitor (DPP-4i) treatment received versus no treatment (Kaplan–Meier plots) for the composite outcome of cardiovascular (CV) death or heart failure hospitalization (hHF) (**a**) and for CV death (**b**), both occurring after the first within-trial nonfatal myocardial infarction (MI) (defining day 0 on the x-axis). On-treatment sensitivity analysis

At the time of the first nonfatal MI, 249 (42%) participants were taking a DPP-4i and 341 (58%) were not. There was no significant difference in the composite outcome of CV death or hHF for those treated or not treated with a DPP-4i (Fig. 2a and Table 3) for either unadjusted analyses (HR 0.91, 95% CI 0.62–1.34, P = 0.63) or adjusted analyses (HR 0.95, 95% CI 0.64–1.43, P = 0.82). All results were consistent with those for the intention-to-treat analyses, although CV deaths were numerically less in those treated with a DPP4i (HR 0.75). Results were also consistent when first fatal MI was included in the analysis (Additional file 1: Table S3, Figure S2).

**Table 3:** Cardiovascular outcomes occurring after a first within-trial nonfatal myocardial infarction in those pretreated or not pretreated with a dipeptidyl peptidase-4 inhibitor (DPP-4i) (on-treatment sensitivity analysis)

	DPP-4i treated n = 249		Not DPP-4i treated n = 341		Unadjusted hazard ratio (95% CI)	P-value	Adjusted hazard ratio (95% CI)	P-value
	No. (%)	Events per 100 patient-years	No. (%)	Events per 100 patient-years				
Cardiovascular death or hospitalization for heart failure	45 (18.1)	11.9	62 (18.2)	13.3	0.91 (0.62–1.34)	0.63	0.95 (0.64–1.43)	0.82
Cardiovascular death	25 (10.0)	6.2	40 (11.7)	8.1	0.78 (0.47–1.29)	0.34	0.75 (0.44–1.26)	0.27
Hospitalization for heart failure	27 (10.8)	7.2	30 (8.8)	6.4	1.15 (0.68–1.94)	0.60	1.34 (0.77–2.33)	0.31
New onset heart failure	16 (6.4)	4.0	20 (5.9)	4.1	1.05 (0.54–2.05)	0.88	1.34 (0.64–2.79)	0.44
Cardiovascular death, hospital admission for heart failure, new heart failure, acute myocardial infarction, stroke or new-onset atrial fibrillation	87 (34.9)	28.8	120 (35.2)	31.8	0.92 (0.70–1.22)	0.56	0.95 (0.71–1.27)	0.72
Further acute myocardial infarction	46 (18.5)	7.2	63 (18.5)	7.3	0.97 (0.66–1.42)	0.89	0.99 (0.67–1.46)	0.95
All-cause death	37 (14.9)	8.9	49 (14.4)	9.9	0.94 (0.61–1.44)	0.77	0.91 (0.58–1.43)	0.68

## Discussion

Although preclinical data provided theoretical support [19–22], these post hoc TECOS analyses found no evidence that treatment with sitagliptin, compared with placebo, given prior to a first within-trial nonfatal MI had any impact on subsequent CV outcomes. Similar results were obtained when previous use of any DPP-4i was examined, and in sensitivity analyses that included fatal as well as nonfatal MIs.

Possible explanations for the discordance between human and animal observations include the following: (1) all TECOS participants had established ASCVD versus the lack of disease in experimental animals; (2) our study had only modest statistical power with just 123 composite outcome events analyzed; (3) experimentally induced MI is typically the consequence of total occlusion of a large coronary vessel, leading to a rather large area of myocardial necrosis, associated with adverse clinical consequences and significant mortality in the animal models—in contrast, spontaneous acute MI in humans is more variable in terms of the size of the relevant coronary vessel and the corresponding size of the subtended myocardium, whether complete occlusion of the coronary occurs, and marked variability in the timing from MI onset to clinical presentation, all of which translates into highly variable sizes of the area at risk, i.e. receiving blood supply from the infarct-related vessel, and of the necrotic area [25, 26]; (4) the doses of sitagliptin used in the animal studies are roughly twofold or more higher [19–22]; and (5) not all TECOS participants may have been adherent with respect to their study medication, and the GLP-1 receptor agonism augmented by DPP-4is does not have the same CV consequences in humans that has been demonstrated in animal studies [15–22]. Our results, however, are supported by negative results reported from a similar analysis of the Liraglutide Effect and Action in Diabetes: Evaluation of Cardiovascular Outcome Results (LEADER) trial examining effects of liraglutide versus placebo pretreatment on CV events following MI occurring during the trial [30].

Controversy persists regarding the effects of DPP-4is on heart failure risk, originating from the observation of an increased risk of hHF with saxagliptin in the Saxagliptin Assessment of Vascular Outcomes Recorded in Patients with Diabetes Mellitus-Thrombolysis in Myocardial Infarction 53 (SAVOR-TIMI) 53 trial [31] with a similar non-significant trend in the Examination of Cardiovascular Outcomes with Alogliptin versus Standard of Care (EXAMINE) trial with alogliptin [32], but no hHF signal observed with sitagliptin [27] or linagliptin [33]. On the other hand, results from observational studies have yielded counter-observations, reporting lower hHF risk associated with DPP-4i use compared with GLP-1 receptor agonists, with no significant difference in patients with a history of heart failure [34], and no difference in the risk of hHF when DPP-4i use was compared with sulfonylurea [35]. If DPP-4i treatment increases heart failure risk, the mechanism remains elusive. By echocardiographic criteria, a trend toward worsening diastolic ventricular function was slowed with sitagliptin treatment [36]. As a potential reason for a heterogeneity in effects between different DPP-4is, a suppression of renal sodium-hydrogen exchanger 3 activity with agents that are excreted in the urine (sitagliptin, alogliptin and linagliptin) has been proposed to protect from DPP-4i–induced heart failure [37]. In the present analysis, in accord with prior results of no heart failure effects of sitagliptin in the overall TECOS cohort, no association between sitagliptin and heart failure events was observed post-MI [7, 8, 27]. Thus, sitagliptin seems to be safe in patients during and after acute MI. Whether this applies to other DPP-4is needs to be studied in dedicated analyses from the respective CV outcomes trials [5, 6, 10]. Along these lines, a meta-analysis of other CV outcomes trials with DPP-4is (e.g. SAVOR TIMI-53 [5], EXAMINE [6], CArdiovascular safety and Renal Microvascular outcomE study with LINAgliptin [CARMELINA] [9, 10], and CARdiovascular Outcome Trial of LINAgliptin Versus Glimepiride in Type 2 Diabetes [CAROLINA] [38]) could provide further clarification.

Limitations of the present analyses include the non-randomized selection of the subset with MI for analysis [7, 8]. In addition, incomplete adherence to randomized treatment that could have occurred selectively post-MI could further confound comparative analyses. These analyses had limited power given the relatively few patients with MI with subsequent outcomes of interest. However, this data set is larger than most available with an ability to explore such associations.

## Conclusions

In summary, these post hoc analyses of data from TECOS participants who had type 2 diabetes and ASCVD do not support the preclinically derived hypothesis that DPP-4i treatment prior to an MI can reduce the subsequent risk of CV death or hHF.

## Supplementary information

**Additional file 1: Table S1.** Factors included in adjustment models for each clinical endpoint. **Table S2.** Cardiovascular outcomes occurring after a first within-trial fatal or nonfatal myocardial infarction in those randomized previously to sitagliptin or placebo treatment (intention-to-treat analysis). **Table S3.** Cardiovascular outcomes occurring after a first within-trial fatal or nonfatal myocardial infarction in those pretreated or not pretreated with a DPP-4i (on-treatment sensitivity analysis). **Figure S1.** Unadjusted event curves by randomized assignment to sitagliptin or placebo (Kaplan–Meier plots) for the composite outcome of cardiovascular death or heart failure hospitalization (A) and for cardiovascular death (B), both occurring after the first within-trial myocardial infarction during the TECOS trial (defining day 0 on the x-axis). Intention-to-treat analysis. **Figure S2.** Unadjusted event curves by treatment received, DPP-4i versus no DPP-4i, Kaplan-Meier plots for the composite outcome of cardiovascular death or heart failure hospitalization (A), and for cardiovascular death (B), both occurring after the first nonfatal within-trial myocardial infarction (defining day 0 on the x-axis). On-treatment sensitivity analysis.

## Data Availability

Requests to access the data for this study from qualified researchers trained in human subject confidentiality protocols may be submitted at dcri.org/data-sharing.

## Abbreviations

ASCVD: atherosclerotic cardiovascular disease
CV: cardiovascular
DPP-4is: dipeptidyl peptidase-4 inhibitors
hHF: hospitalization for heart failure
MI: myocardial infarction
TECOS: Trial Evaluating Cardiovascular Outcomes with Sitagliptin
