# Supplementary material for: Sitagliptin does not reduce the risk of cardiovascular death or hospitalization for heart failure following myocardial infarction in patients with diabetes: observations from TECOS
Source: Cardiovasc Diabetol. 2019 Sep 3;18:116. doi: 10.1186/s12933-019-0921-2 (PMC6719352; doi:10.1186/s12933-019-0921-2)
Supplement: Supplementary file 1 — Additional file 1: Table S1. Factors included in adjustment models for each clinical endpoint. Table S2. Cardiovascular outcomes occurring after a first within-trial fatal or nonfatal myocardial infarction in those randomized previously to sitagliptin or placebo treatment (intention-to-treat analysis). Table S3. Cardiovascular outcomes occurring after a first within-trial fatal or nonfatal myocardial infarction in those pretreated or not pretreated with a DPP-4i (on-treatment sensitivity analysis). Figure S1. Unadjusted event curves by randomized assignment to sitagliptin or placebo (Kaplan–Meier plots) for the composite outcome of cardiovascular death or heart failure hospitalization (A) and for cardiovascular death (B), both occurring after the first within-trial myocardial infarction during the TECOS trial (defining day 0 on the x-axis). Intention-to-treat analysis. Figure S2. Unadjusted event curves by treatment received, DPP-4i versus no DPP-4i, Kaplan-Meier plots for the composite outcome of cardiovascular death or heart failure hospitalization (A), and for cardiovascular death (B), both occurring after the first nonfatal within-trial myocardial infarction (defining day 0 on the x-axis). On-treatment sensitivity analysis. [file 12933_2019_921_MOESM1_ESM.docx]

**Additional file 1**

**Table S1.** Factors included in adjustment models for each clinical endpoint

|  | **CV Death or HF Hospitalization** | **CV Death** | **Hospitalization for HF** | **New Onset HF** | ***Composite Outcome** | **New MI** | **All-Cause Mortality** |
| --- | --- | --- | --- | --- | --- | --- | --- |
| Sex | **X** | **X** |  |  | **X** | **X** | **X** |
| Current smoker | **X** | **X** |  |  | **X** | **X** | **X** |
| Race |  |  |  |  | **X** | **X** |  |
| Age | **X** | **X** | **X** | **X** | **X** | **X** | **X** |
| Pulse pressure |  |  |  |  | **X** | **X** |  |
| Body mass index |  |  |  |  | **X** | **X** | **X** |
| Weight | **X** |  | **X** | **X** | **X** | **X** |  |
| Height | **X** |  | **X** | **X** | **X** | **X** |  |
| Systolic blood pressure | **X** | **X** |  |  |  |  |  |
| UACR | **X** | **X** | **X** | **X** | **X** | **X** | **X** |
| Estimated glomerular filtration rate |  | **X** |  |  | **X** | **X** | **X** |
| Hemoglobin | **X** | **X** | **X** | **X** | **X** | **X** | **X** |
| Low density lipoprotein |  |  |  |  | **X** | **X** | **X** |
| Hemoglobin A1c | **X** | **X** | **X** | **X** | **X** | **X** | **X** |
| Duration of diabetes | **X** | **X** | **X** | **X** | **X** | **X** | **X** |
| *History of:* |  |  |  |  |  |  |  |
| HF | **X** | **X** |  |  | **X** | **X** | **X** |
| COPD | **X** | **X** | **X** | **X** | **X** | **X** | **X** |
| Cerebrovascular disease | **X** | **X** | **X** | **X** | **X** | **X** | **X** |
| Peripheral artery disease | **X** | **X** | **X** | **X** | **X** | **X** | **X** |
| Cardiovascular disease | **X** | **X** | **X** | **X** | **X** | **X** | **X** |
| Atrial fibrillation or flutter | **X** | **X** | **X** | **X** | **X** | **X** | **X** |
| Time by Age# | **X** | **X** |  |  | **X** |  | **X** |
| Time by COPD# | **X** |  | **X** | **X** |  |  |  |
| Time by atrial fibrillation or flutter# | **X** |  | **X** | **X** |  |  |  |
| Time by UACR# | **X** |  | **X** | **X** |  |  |  |

*CV death, hospitalization for HF, new HF, acute MI, stroke or new-onset atrial fibrillation.

#These factors did not meet the proportional hazards assumption. Therefore, the interaction of each factor with time to the event was included in the model to allow for a change in hazard over time.

COPD=chronic obstructive pulmonary disorder, UACR=urine albumin to creatinine ratio, CV=cardiovascular, HF=heart failure, MI=myocardial infarction.

**Table S2.** Cardiovascular outcomes occurring after a first within-trial fatal or nonfatal myocardial infarction in those randomized previously to sitagliptin or placebo treatment (intention-to-treat analysis)

|  | | **Sitagliptin**  **n=300** | | | **Placebo**  **n=316** | | |  | |  | |  | |  | |
| --- | --- | --- | --- | --- | --- | --- | --- | --- | --- | --- | --- | --- | --- | --- | --- |
|  | **No. (%)** | | **Events per 100 patient-years** | **No. (%)** | | **Events per 100 patient-years** | **Unadjusted hazard ratio (95% CI)** | | **P-value** | | **Adjusted**  **hazard ratio (95% CI)** | | **P-value** | |  |
| Cardiovascular death or hospitalization for heart failure | 65 (21.7) | | 15.6 | 58 (18.4) | | 13.5 | 1.17 (0.82–1.67) | | 0.38 | | 1.22 (0.84–1.77) | | 0.29 | |  |
| Cardiovascular death | 41 (13.7) | | 9.2 | 40 (12.7) | | 8.8 | 1.07 (0.69–1.66) | | 0.75 | | 1.08 (0.68–1.70) | | 0.75 | |  |
| Hospitalization for heart failure | 31 (10.3) | | 7.5 | 26 (8.2) | | 6.1 | 1.26 (0.75–2.12) | | 0.39 | | 1.39 (0.80–2.42) | | 0.24 | |  |
| New onset heart failure | 19 (6.3) | | 4.3 | 17 (5.4) | | 3.8 | 1.25 (0.64–2.43) | | 0.51 | | 1.49 (0.72–3.09) | | 0.28 | |  |
| Cardiovascular death, hospital admission for heart failure, new heart failure, acute myocardial infarction, stroke or new-onset atrial fibrillation | 115 (38.3) | | 35.1 | 108 (34.2) | | 30.7 | 1.15 (0.88–1.49) | | 0.31 | | 1.19 (0.91–1.57) | | 0.21 | |  |
| Further acute myocardial infarction | 54 (18.0) | | 7.2 | 55 (17.4) | | 6.9 | 1.03 (0.70–1.50) | | 0.89 | | 1.00 (0.68–1.48) | | 0.99 | |  |
| All-cause death | 57 (19.0) | | 12.5 | 45 (14.2) | | 9.9 | 1.32 (0.89–1.95) | | 0.17 | | 1.31 (0.87–1.97) | | 0.19 | |  |

**Table S3.** Cardiovascular outcomes occurring after a first within-trial fatal or nonfatal myocardial infarction in those pretreated or not pretreated with a DPP-4i (on-treatment sensitivity analysis)

|  | | | **Not Treated with DPP-4i**  **n=362** | | | | | **DPP-4i Treated**  **n=253** | | | |  | | | | | | | |  |
| --- | --- | --- | --- | --- | --- | --- | --- | --- | --- | --- | --- | --- | --- | --- | --- | --- | --- | --- | --- | --- |
|  | | **No. (%)** | | **Events per 100 patient-years** | | **No. (%)** | | | | **Events per 100 patient-years** | **Unadjusted hazard ratio (95% CI)** | | **P-value** | | **Adjusted** **hazard ratio (95% CI)** | | **P-value** | |  |  |
| Cardiovascular death or hospitalization for heart failure | 75 (20.7) | | | | 16.04 | | 47 (18.6) | | 12.45 | | 0.79 (0.55–1.14) | | | 0.21 | | 0.82 (0.56–1.20) | | 0.30 | | |
| Cardiovascular death | 53 (14.6) | | | | 10.73 | | 27 (10.7) | | 6.64 | | 0.65 (0.41–1.03) | | | 0.07 | | 0.61 (0.38–0.99) | | 0.05 | | |
| Hospitalization for heart failure | 30 (8.3) | | | | 6.42 | | 27 (10.7) | | 7.15 | | 1.15 (0.68–1.94) | | | 0.60 | | 1.34 (0.77–2.33) | | 0.30 | | |
| New onset heart failure | 20 (5.5) | | | | 4.08 | | 16 (6.3) | | 3.97 | | 1.05 (0.54–2.05) | | | 0.88 | | 1.34 (0.64–2.79) | | 0.44 | | |
| Cardiovascular death, hospital admission for heart failure, new heart failure, acute myocardial infarction, stroke or new-onset atrial fibrillation | 133 (36.7) | | | | 35.26 | | 89 (35.2) | | 29.44 | | 0.85 (0.65–1.12) | | | 0.25 | | 0.88 (0.66–1.16) | | 0.35 | | |
| Further acute myocardial infarction | 63 (17.4) | | | | 6.97 | | 46 (18.2) | | 7.10 | | 1.01 (0.69–1.47) | | | 0.98 | | 1.03 (0.69–1.52) | | 0.90 | | |
| All-cause death | 62 (17.1) | | | | 12.49 | | 39 (15.4) | | 9.42 | | 0.79 (0.53–1.18) | | | 0.25 | | 0.76 (0.50–1.16) | | 0.20 | | |

**Figure S1**

**A**

**
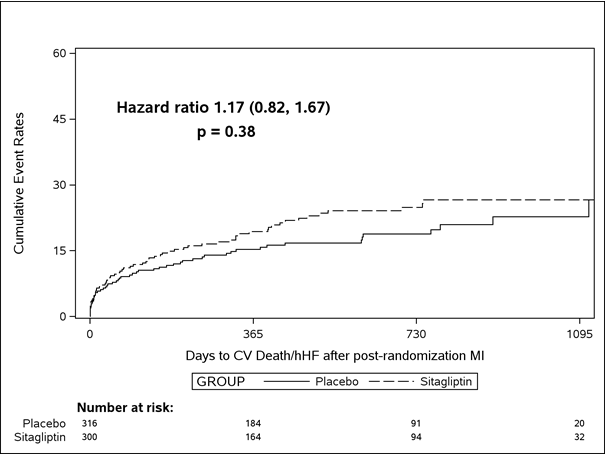
**

**B**


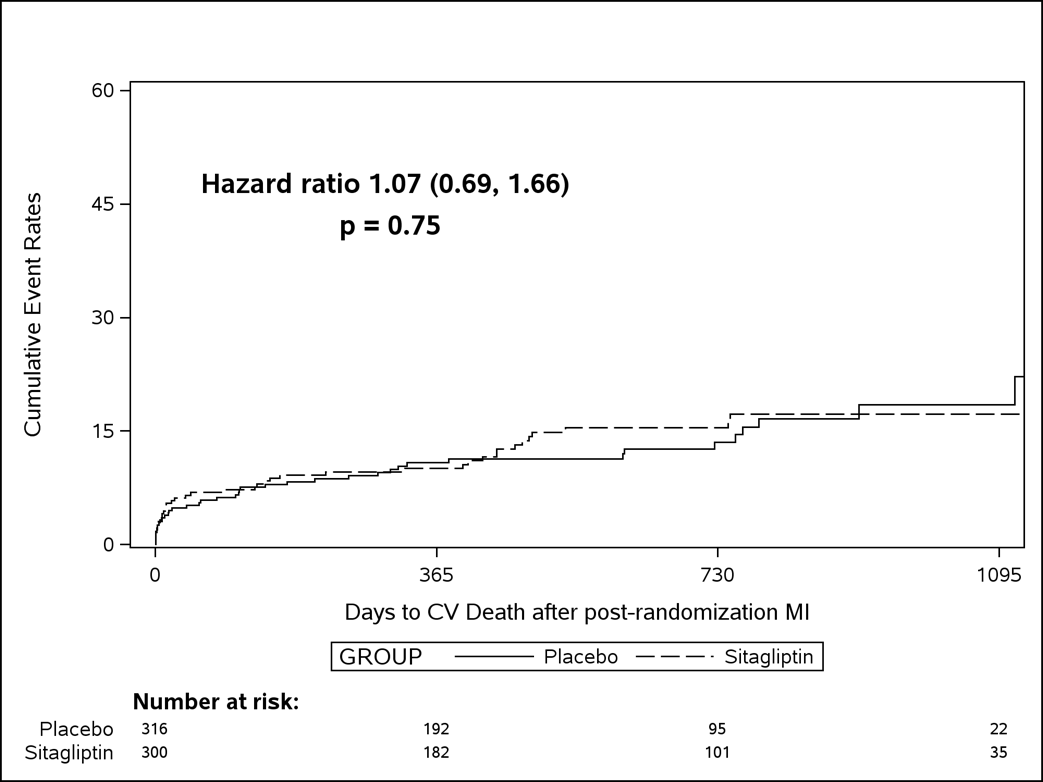


**Hazard ratio 1.07 (0.69, 1.66)
p = 0.75**

Unadjusted event curves by randomized assignment to sitagliptin or placebo (Kaplan-Meier plots) for the composite outcome of cardiovascular death or heart failure hospitalization (A) and for cardiovascular death (B), both occurring after the first within-trial myocardial infarction during the TECOS trial (defining day 0 on the x-axis). Intention-to-treat analysis.

**Figure S2**

**A**


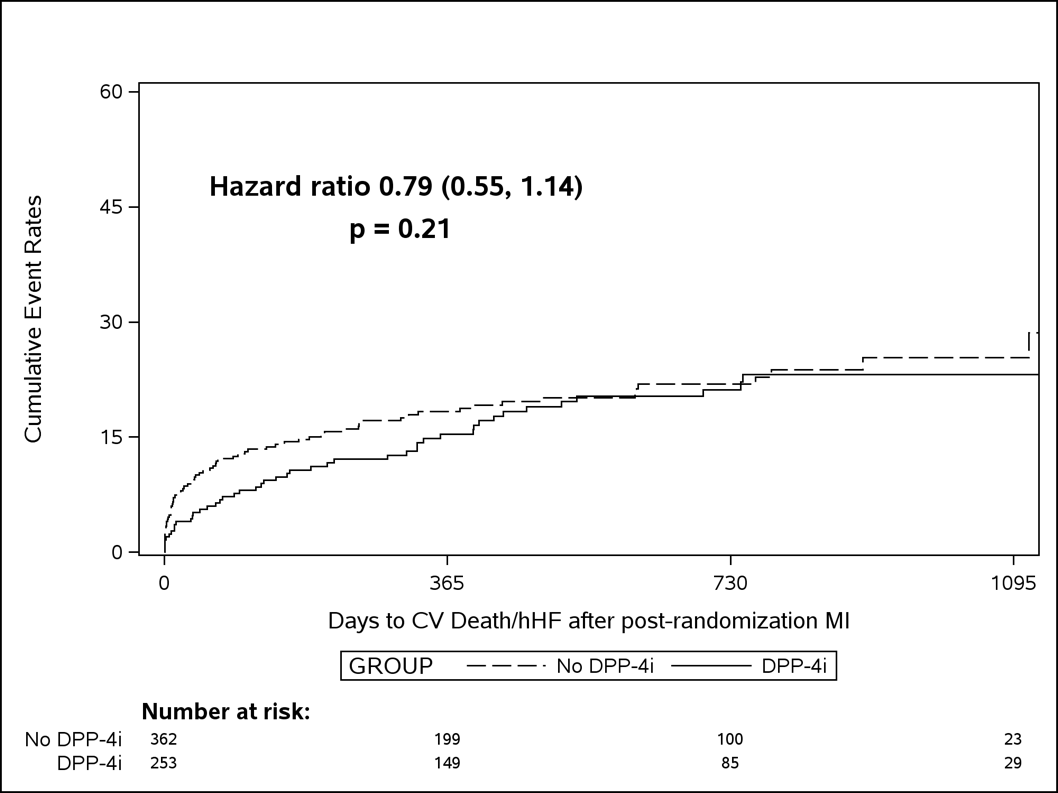


**B**


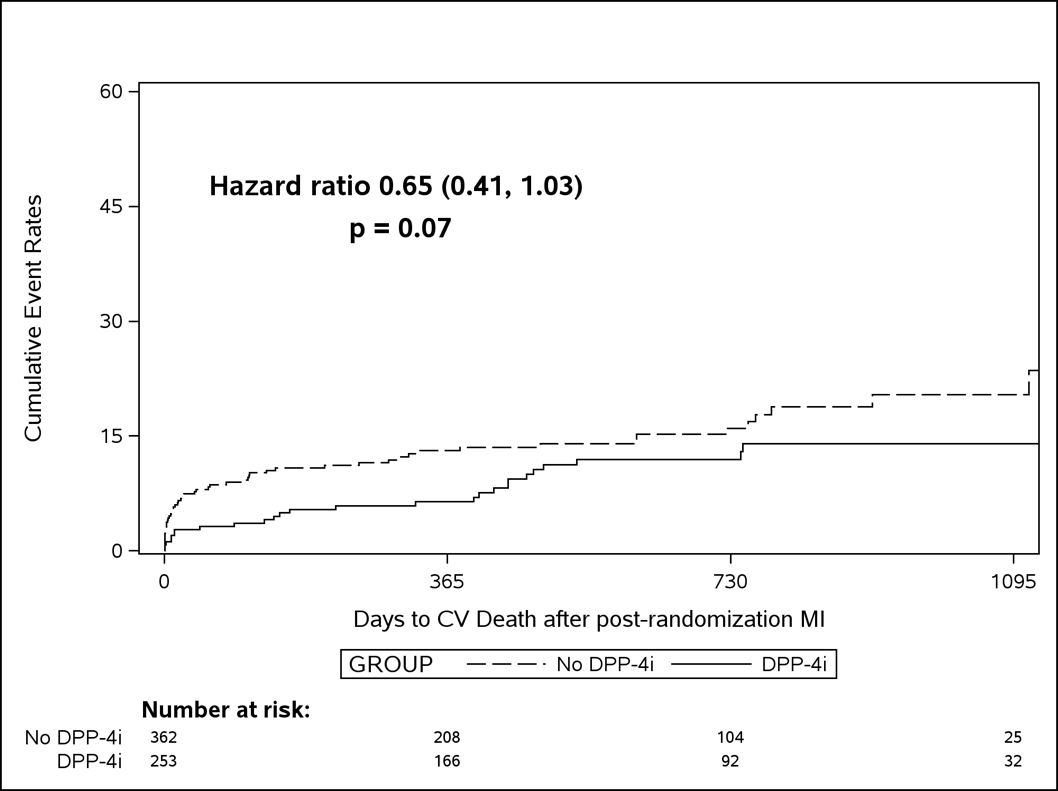


Unadjusted event curves by treatment received, DPP-4i versus no DPP-4i, Kaplan-Meier plots for the composite outcome of cardiovascular death or heart failure hospitalization (A), and for cardiovascular death (B), both occurring after the first nonfatal within-trial myocardial infarction (defining day 0 on the x-axis). On-treatment sensitivity analysis.
